# Enhancing the Production of H_2_ and Volatile Fatty Acids by Fungal Pretreatment of Invasive Macroalgae () Followed by Dark Fermentation

**DOI:** 10.1021/acs.energyfuels.5c01245

**Published:** 2025-06-30

**Authors:** Pedro Fernández-Medina, Cristina Agabo, Ana Blandino, Luis I. Romero-García, Carlos J. Álvarez-Gallego

**Affiliations:** Department of Chemical Engineering and Food Technology, Faculty of Sciences, (Wine and Agri-Food Research Institute-IVAGRO and International Campus of Excellence-ceiA3), University of Cadiz, Republic Saharawi Avenue, P.O. Box No. 40, 11510 Puerto Real, Cádiz, Spain

## Abstract

The blue economy promotes the sustainable use of marine resources, such as algae, for socioeconomic development. This study aims to increase the production of hydrogen (H_2_) and volatile fatty acids (VFA) through the dark fermentation of the invasive macroalga . Batch experiments were conducted to identify the optimal operating conditions, with the following parameters being varied: inoculum-substrate ratio (IS) (0.43, 0.25, and 0.11 v/v); initial pH (5.5, 6.5, and 7.5); and algal suspension concentration (4, 6, 8, and 10% w/v). Additionally, the impact of biological pretreatment using was assessed. This was performed via solid-state fermentation (SSF) at two fungal inoculum concentrations (10^7^ and 10^8^ cel/g_biomass_) and two incubation times (5 and 8 days). The highest H_2_ yield (9.49 mL_H_2__/g_biomass_) was obtained after 8 days of pretreatment with 10^7^ cel/g_biomass_, whereas the greatest TVFA production (213 mg of acetic acid equivalents/g_biomass_) occurred after 5 days using 10^8^ cel/g_biomass_. These results suggest that biological pretreatment can enhance the performance of dark fermentation and that the latter condition is most suitable for VFA-oriented biorefineries.

## Graphical abstract

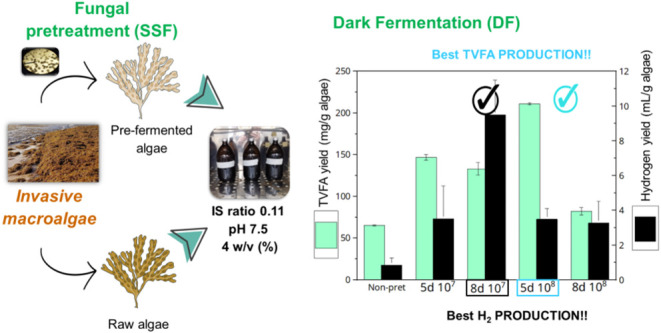

## 1 Introduction

 was first detected in the Strait of Gibraltar in 2015; from then, it massively proliferated until it was recognized as an invasive species less than a year after its arrival.^1^ This rapid proliferation was due to its high growth rate and effective biological mechanisms to ensure its survival against predators,^2^ which caused major changes in the marine ecosystem.^2^ Nowadays, there is the appearance of , 7 years ago, which continues to cause a great impact not only along the coast of the Spanish region of Andalusia but also in other regions such as Comunitat Valenciana^3^ and even in other countries, increasing both the magnitude and range of the problem.^4^

The blue economy aims to use marine resources for socioeconomic purposes in a sustainable mode.^5^ Because of its importance, numerous studies exist in which valuable products such as biosorbents, biofuels, or fertilizers are obtained from marine products.^6^ Traditionally, fishing discards have been one of the main raw materials to obtain these valuable products, although recently, algal biomass has also emerged to be useful for this purpose.

Using macroalgae as a substrate to obtain valuable products relies on their chemical composition, which exhibits high water (70–90%) and mineral (10–15%) contents, with a minimal lignin proportion.^7^ Additionally, some species show a substantial amount of carbohydrates, making macroalga a valuable raw material for fermentation processes. Nonetheless, the polysaccharide composition is variable depending on the species. In particular, is classified as a brown seaweed that contains approximately 60% carbohydrates on a dry weight basis and is mainly composed of alginate, fucoidan, and cellulose.^8^

Despite this high carbohydrate content, the majority of them are structural polymers that are difficult to biodegrade without pretreatment. Because of this, a large number of pretreatments have been carried out on algal biomass, including thermochemical,^9–11^ microwave,^12,13^ ultrasound,^14,15^ and others,^16^ to increase its degradability before any bioprocess.

In this sense, dark fermentation (DF) is considered a cheap and easily implemented bioprocess for the production of two high-value-added products: volatile fatty acids (VFA) and H_2_.^17^ In this process, the yields of the products are closely related to the type of substrate, inoculum, and operation conditions (such as temperature or pH), which are normally optimized.^18^ However, it is difficult for fermentative acidogenic microorganisms to consume the intracellular components of the macroalga, necessitating the application of an appropriate pretreatment.^11^ In this sense, only a few studies have tested different pretreatments to improve the DF of brown macroalgal biomass, with thermal and thermochemical treatments being the most preferred ones to enhance the solubilization of carbohydrate polymers into soluble sugars.^9–13^

In this sense, Yin and Wang^11^ tested different thermochemical pretreatments before DF of , obtaining the maximal increase of H_2_ and VFA yields with heat-base pretreatment (2% NaOH, 121 °C, 30 min), resulting in increases of 2.7 and 1.7 times, respectively. In a posterior study^19^ by these authors, microwave-assisted acid pretreatment was used, testing different concentrations of H_2_SO_4_ (0, 0.5, 1, and 2% (v/v)). The best conditions were 1% H_2_SO_4_, 140 °C, and increase in the H_2_ yield (mL_H_2__/g_biomass_) and VFA yield (mg_VFA_/g_biomass_) by 1.9 and 4.4 times, respectively, with respect to the non-pretreated seaweed. Fernández-Medina et al.^12^ used the microwave to pretreat , finding an H_2_ yield increase of almost 6 times at 200 °C and a TVFA yield increase of 3 times at 220 °C, both compared to non-pretreated alga. Finally, Chen et al.^16^ studied the effect of γ irradiation on H_2_ production using and obtained 1.4–1.7 times H_2_ yield and 1.3–1.0 times AGV yield by using an irradiation of 10–20 kGy compared to not irradiated algae. Although these pretreatments are effective, many may cause negative environmental impacts due to the use of polluting chemical compounds (i.e., thermochemical) and/or the consumption of high energy levels (i.e., microwave).^20^

Therefore, the least energy-intensive and environmentally friendly pretreatments could provide a valuable alternative to conventional ones for these processes. Among them, one of the most promising is solid-state fermentation (SSF) with fungi,^21^ which has been successfully applied to different biomasses to improve the DF process.^22^ During SSF, the fungus secretes enzymes that hydrolyze the constituent biopolymers of the biomass. Moreover, SSF offers several advantages over submerged fermentation, including low water consumption and reduced use of other reagents, such as antifoam agents. It can be applied under semisterile conditions, resulting in higher enzymatic yields, temperature and pH stability, and shorter fermentation times. This leads to lower operating costs.^23^ In this sense, it has been reported that some hydrolytic enzymes, like laminarinase and alginate lyase, are expressed during fungal SSF of brown seaweed, favoring the biomass solubilization in the subsequent DF process.^24,25^

The current work established the most suitable operating conditions for obtaining volatile fatty acids and H_2_ from the DF of seaweed . In addition, the improvement in VFA and H_2_ yields by using biologically pretreated seaweed with the fungus was analyzed.

## 2 Materials and Methods

### 2.1 Seaweed Biomass

Seaweed was harvested from a beach located in the south of Spain (Tarifa, 36°00′44.1″N 5°35′53.9″W) in April 2021. The collected samples were subjected to a 24 h washing process to eliminate any residual sand or salt. This washing procedure involved continuous rinsing with tap water. After the 24 h, the conductivity of the water discharged from the macroalga washing tanks was found to be comparable to that of the inlet water, indicating the completion of salt release. The seaweed was then naturally air-dried for 48 h in a greenhouse until it reached 13% total humidity. Once dried, the biomass was ground to a particle size of less than 1 mm. The physicochemical characteristics have been described in previous studies.^12^

### 2.2 Biological Pretreatment

Pretreatment was conducted via solid-state fermentation (SSF) with the fungus 2B.361 U2/1, which is a sequential mutant of NRRL 3312. was grown in Petri dishes at 30 °C for 5 days on a synthetic medium similar to that used in previous studies,^24^ except that sodium alginate was used instead of pectin. Alginate is indeed one of the most abundant biopolymers in brown seaweed. Exposing the fungal strain continuously to alginate in multiple passes could help select individuals with more specific alginate lyase production, ensuring that proper growth is transmitted to new generations and making the strain more productive and adapted to the new substrate.

Fungal spores were harvested by lightly scraping the plate surface with 2 mL of a 0.9 w/v NaCl solution. After collection, the number of spores in suspension was determined by optical microscopy (Leica DME) using an improved Neubauer chamber, resulting in a final concentration of 10^8^ spores/mL.

Biological pretreatment trials were conducted in triplicate within sterilized 1L Erlenmeyer flasks using the fungus in the same ratio of nutrient solution as used in a previous work.^26^ For this purpose, 20 g of algae and 60 mL of Mendel’s solution were sterilized by autoclaving at 120 °C for 20 min in an autoclave type SELECTA, followed by cooling and inoculation with 20 mL of spore suspension at 10^7^ and 10^8^ spores/mL. The flasks were incubated in an incubator (Incu-Line VWR) under static conditions at 30 °C for 5 and 8 days and inoculated with 10^7^ and 10^8^ spores/g_biomass_. The assays were named as “5d 107”, “5d 108”, “8d 107”, and “8d 108”.

### 2.3 Enzyme Extraction and Activities

Fermented in a 250 mL Erlenmeyer flask were suspended in 0.1% v/v Tween 80 at a ratio of 1:20 (w/v) and incubated in a rotary shaker (MAXQ6000, Thermo Scientific) for 30 min at 4 °C and 150 rpm. Then, the suspensions were centrifuged (Eppendorf 5810R) at 10,000 rpm and 4 °C for 10 min, and the supernatant liquor (the enzymatic extract) was used to measure the cellulase (FPase, EC 3.2.1.91), alginate lyase (EC 4.2.99.4), and total reducing sugars (TRS), according to the previous research work.^24^ Laminarase activity (EC 3.2.1.6) was also determined through modification of an existing protocol, using 0.3% laminarin in 50 mM potassium phosphate buffer (pH 6.5) as substrate.^27^ The enzymatic reaction was performed by incubating 0.25 mL of the substrate and 0.25 mL of enzyme solution for 20 min at 30 °C. FPase/alginate lyase/laminarinase activity was defined as the amount of enzyme required to release 1 μmol of reducing sugars per minute at defined assay conditions.

### 2.4 Dark Fermentation

DF assays were conducted in triplicate using 250 mL vials in batch mode. Each vial was filled with the alga suspension and the inoculum, with a total working volume of 150 mL. The remaining space in the vial was occupied by the biogas produced during the fermentation.

The inoculum used in the DF tests consists of the effluent from an acidogenic reactor systematically operated at pH 5.5, 55 °C, and a hydraulic retention time (HRT) of 5 days. This reactor followed a semicontinuous daily feeding regime using exhausted sugar beet pulp (ESBP) from a nearby sugar factory located in Jerez de la Frontera, Spain (AB Sugar). The physicochemical characteristics of the effluent from the reactor are shown in Table 1.

**Table 1:** Main Physicochemical Features of DF Reactor Effluent for a Significance of *p* < 0.05 Expressed in Dry Basis

parameter	units	value
total solids	g/kg	478 ± 5.72
volatile solids	g/kg	282 ± 7.75
total alkalinity	g/kg	39.7 ± 1.15
soluble chemical oxygen demand	g/kg	28.7 ± 0.84
total polyphenols	g/kg	0.82 ± 0.02
total volatile fatty acids	g/kg	27.1 ± 1.15

For obtaining the inoculum, the acidogenic reactor effluent was centrifuged at 50 g for 10 min, reducing the solid content to 8.44 g/kg volatile solids as described in previous studies.^12^ This step was taken to reduce the amount of organic matter provided by the inoculum, enhancing the early algal biomass consumption through DF tests.

Dark fermentation assays to determine the optimal operating conditions (initial pH, alga-inoculum ratio, and algal suspension concentration) were carried out using non-pretreated seaweed. For this purpose, the DF trials were undertaken at different inoculum-substrate­(alga) ratios (IS (% v/v): 30/70 = 0.43, 20/80 = 0.25, and 10/90 = 0.11 v/v), initial pH values (pH: 5.5, 6.5, and 7.5), and solid loading of algae in the substrate (SL (% w/v): 4, 6, 8, and 10). Vials containing only inocula were used as the inoculum control.

Once the optimal fermentation operating conditions had been established, DF trials were carried out under these conditions using biologically pretreated macroalga. For these assays, additional vials containing non-pretreated alga were also used as the non-pretreated seaweed control.

For the development of the fermentations, first the algal biomass suspended in distilled water was mixed with the acidogenic inoculum and pH was adjusted to the established value. After that, vials were filled with the mixture in a working volume of 150 mL. After the vials were sealed, they were incubated at static conditions at 55 °C for 9 days. Throughout the 3 initial days, headspace overpressure was daily quantified by a digital pressure gauge (Omega HPP350) and the generated biogas was discharged after determining its composition. Subsequent measurements were performed only on days 6 and 9. The bio-H_2_ yield was calculated as the ratio of cumulative H_2_ production, expressed in mL at STP. In addition, control parameters were analyzed at the beginning and at the end of the fermentation process as described in Section 2.5. The values of these parameters presented in the results correspond to the exclusive contribution of the macroalga in the dark fermentation tests, as the possible contribution of the inoculum in these assays was subtracted by using the data obtained from the inoculum control described above.

### 2.5 Analytical Methods

Most of the analytical techniques used were performed in accordance with the APHA-AWWA-WPCF standard methods.^28^ Accordingly, the following determinations from this reference were applied: total solids (TS) by 2540B, volatile solids (VS) by 2540E, soluble chemical oxygen demand (sCOD) by 5220C, dissolved organic carbon (DOC) by 5310B, total alkalinity (TA) by 2320B, and pH by 4500H^+^. Additionally, the quantification of total polyphenols (TP) was performed according to the Folin-Ciocalteu method, as outlined in the literature,^29^ while TRS was determined using the 3,5-dinitrosalicylic acid (DNS) method, based on the absorbance measurement at 540 nm in a microplate reader.^30^ Volatile fatty acids (VFAs) were analyzed via gas chromatography on a Shimadzhu GC-2010 equipped with a flame ionization detector and a capillary column (Nukol Supelco Merck kGaA), featuring a diameter of 0.25 mm and a length of 30 m. H_2_ was employed as both the carrier gas and the detector fuel at a flow rate of 50 mL/min. Besides, synthetic air and nitrogen served as the oxidizing and makeup gases, respectively, at flow rates of 400 and 30 mL/min.^31^ Biogas composition was analyzed via gas chromatography, using a Shimadzhu GC-2014 equipped with a thermal conductivity detector and a packed column with a 0.3 cm diameter and a 3 m long molecular sieve packing (Carbosieve Supelco Merck kGaA). Nitrogen served as a carrier gas at a flow rate of 50 mL/min.

Centrifugation at 3200 g for 10 min (Orto Alresa Consul 21 R), followed by filtration to a particle size of 0.47 μm (Hahnemühle, GF52 glass fiber filter), was applied to the samples for DOC, sCOD, TP, and VFAs analyses. In the case of VFA measurement, an additional filtration step was included using a 0.22 filter (Scharlab, PTGE syringe filter). DOC analysis was conducted using an Analytik-Jena multi N/C 3100.^31^

## 3 Results and Discussion

### 3.1 Biological Pretreatment

The TRS and the enzyme activities measured in the enzymatic extracts obtained from biologically pretreated seaweed under different conditions (fermentation time or inoculum size) are shown in Figure 1.

**Figure 1:**
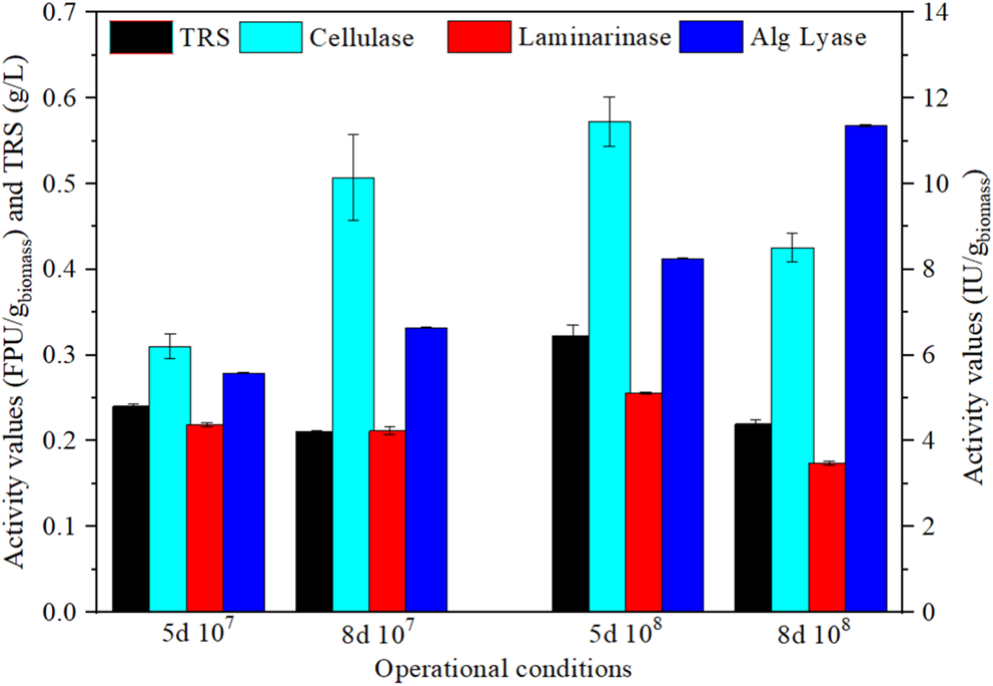
TRS and enzymatic activities measured under different biological conditions. TRS (g/L), black bars; cellulase activity (FPU/g_biomass_), light blue bars; laminarinase activity (IU/g_biomass_), red bars; and alginate lyase activity (IU/g_biomass_), dark blue bars.

In the case of TRS and cellulase as FPase (Figure 1), the highest values were reached at 5 days of 10^8^ cel/g_biomass_: 0.33 ± 0.01 g_TRS_/L and 0.57 ± 0.03 FPU/g_biomass_, respectively. However, for the same concentration of inoculum (10^8^ cel/g_biomass_) but increased time (8 days), the TRS and FPU were reduced until 0.22 ± 0.01 g_TR_S/L and 0.43 ± 0.02 g FPU/g_biomass_. In this sense, this cellulase activity is important to degrade the cellulose fibrils in the inner layer of the cell wall.^32^ However, the enzymes in charge of breaking the macroalgae outer layer showed a higher expression (10 times higher). For example, the storage sugar laminarin was degraded by laminarinase, which was optimal at 5d 10^8^ cel/g_biomass_ (5.12 ± 0.03 IU/g_biomass_). On the other hand, alginates, the principal sugar constituent of^8^ present in the outer matrix, can be broken down by alginate lyase, which was highly expressed, obtaining 8.25 ± 0.01 at 5d 10^8^ cel/g_biomass_ and even 11.4 ± 0.04 IU/g_biomass_ at 8d 10^8^ cel/g_biomass_. Thus, in general, at a proper concentration of spores (10^8^ cel/g_biomass_), enlarging the time of pretreatment of at the SSF of until 8 days is not favorable for increasing sugar-releasing and degrading cell-wall sugar enzymes’ expression.

### 3.2 Optimization of the Dark Fermentation Operational Conditions

The solubilization of organic matter, expressed as ΔsCOD/initial vs (mg O_2_/L)/(g vs/L), and H_2_ yields (mL_H_2__/g_biomass_) and TVFA yields, expressed as equivalent units of acetic acid (mg_H–Ac_/g_biomass_) obtained after the 9 days of dark fermentation from non-pretreated , are shown in Table 2. These results correspond to the different conditions of dark fermentation tested, named with their respective IS:pH:SL values.

**Table 2:** Results Obtained in the DF Tests Applied to Non-Pretreated Macroalga for All of the Conditions Studied over Inoculum Control, Using Significance *p* < 0.05

condition (IS:pH:SL)	ΔsCOD/initial vs (mg O_2_/L)/(g vs/L)	H_2_ yield (mL_H_2__/g_biomass_)	TVFA yield (mg_H–Ac_/g_biomass_)
0.43:7.5:4	47.4 ± 26.5	0.00 ± 0.35	27.4 ± 6.86
0.25:7.5:4	70.2 ± 9.33	0.30 ± 0.28	43.7 ± 2.68
0.11:7.5:4	65.1 ± 19.3	0.63 ± 0.04	43.0 ± 5.08
0.11:5.5:4	15.2 ± 24.3	0.85 ± 0.37	56.4 ± 6.35
0.11:6.5:4	45.8 ± 16.9	0.75 ± 0.13	52.5 ± 1.30
0.11:7.5:4	65.1 ± 19.2	1.01 ± 0.34	64.5 ± 3.53
0.11:7.5:6	23.1 ± 42.4	0.86 ± 0.15	65.2 ± 12.3
0.11:7.5:8	54.3 ± 9.93	0.70 ± 0.16	45.0 ± 4.17
0.11:7.5:10	72.8 ± 4.25	0.56 ± 0.15	41.8 ± 1.19

#### 3.2.1 Effect of the Inoculum-Substrate (IS) Ratio

First, three different conditions of IS ratios were studied at pH 7.5 and using a 4% w/v algae suspension. The highest H_2_ and TVFA yields were obtained for IS ratios of 0.25 and 0.11 (Table 2), showing that the dark fermentation process is favored by the increase of the algal biomass content in the medium.

As can be seen in Table 2, when the IS = 0.43 (30% v/v of inoculum), statistically, no significant differences can be observed in the amount of H_2_ produced in the algal suspension test (0.43:7.5:4). This is also reflected in the curves of Net V_H_2__ accumulated (Supporting Information: Figure S1). Nevertheless, for the other two conditions (0.25:7.5:4 and 0.11:7.5:4), the H_2_ production was considerably higher in the assays performed. This effect is more marked for the IS ratio 0.11. This may suggest that at higher acidogenic inoculum volumes fermentative microorganisms tend to degrade the remaining organic matter present in the inoculum itself rather than the algal biomass.

Thus, as the IS ratio decreases, the amount of remaining inoculum organic matter becomes lower, which significantly enhances algal biomass degradation. In previous DF batch studies it was reported that IS ratios higher than 0.17 may substantially decrease the VFA production and hence the H_2_ yield using different substrates.^33–35^ As an example, Cappai et al.^35^ tested different IS ratios from 0.05 to 0.25 using food waste as a substrate, where the optimal IS was 0.14, very similar to that in this study, indicating the importance of IS in the dark fermentation process.

This claim is supported by the increase in solubilization levels for 0.43:7.5:4; a value of 47.4 was obtained, while for 0.25:7.5:4 the value was 70.21 and for 0.11:7.5:4 it was 65.1 (Table 2). These solubilization values may indicate a greater algae solubilization^12^ in the last two study conditions, which implies a better degradation of the seaweed structures.

This effect is also appreciated for TVFA (Figure 2a), whose production was higher at these two conditions, obtaining around 1400 and 1500 mg_H–Ac_/L, respectively, against 770 mg_H–Ac_/L at 0.43:7.5:4 condition, and mainly for acetic acid, which represents more than 82% of the TVFA production, reaching 1149 ± 56 and 1261 ± 81.5 mg_acetic acid_/L, respectively (Figure 2b).

**Figure 2:**
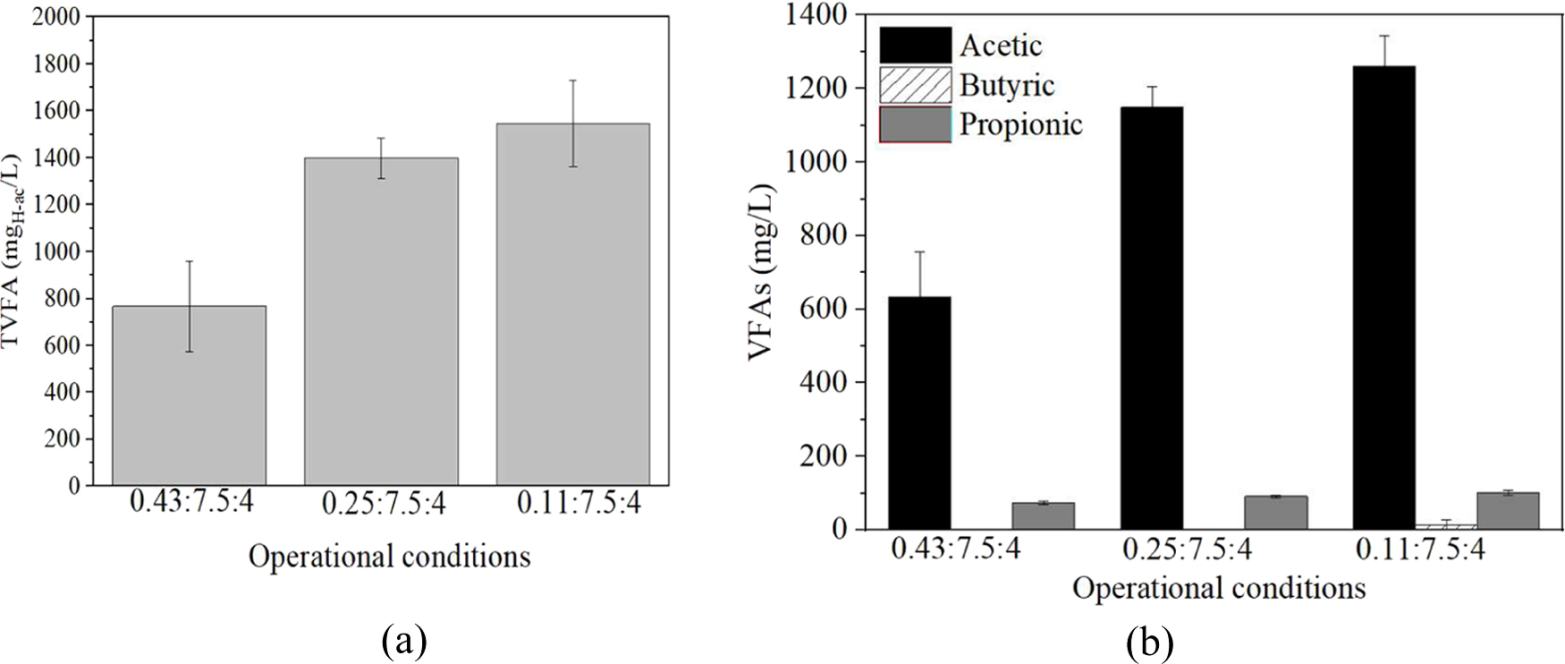
(a) Final TVFA (mg_H‑ac_/L) and (b) their profile (mg/L) for the DF of non-pretreated under different IS conditions. VFAs: acetic acid, black; butyric acid, linear pattern; propionic acid, dark gray. All of the tests were carried out in thermophilic range (*T* = 55 °C), static conditions, pH = 7.5, and SL = 4% w/v.

Taking into consideration these data as well as the TVFA and H_2_ yields obtained for the various IS ratios (Table 2), IS = 0.11 was established as the best ratio tested. It is important to remark that this IS condition is recurrent in the literature using other kinds of brown macroalgae.^19,36^ This was established for the subsequent optimization of the rest of the parameters: pH and percentage of solids in the substrate.

#### 3.2.2 Effect of pH

Regarding Table 2, while for 0.11:6.5:4 and 0.11:7.5:4 the maximum cumulative H_2_ production was almost reached after 1 day of dark fermentation, for 0.11:5.5:4 there was a lag phase during the first days, and the maximum was reached on the third day. So, at pH 7.5 there was a slightly final increase of both production H_2_ volume (Supporting Information: Figure S2) and TVFA production (Table 2), obtaining 5.12 ± 1.35 mL and 2321 ± 127 mg_H–Ac/_L (mainly acetic acid, representing 75% of TVFAs) (Figure 3a,b).

**Figure 3:**
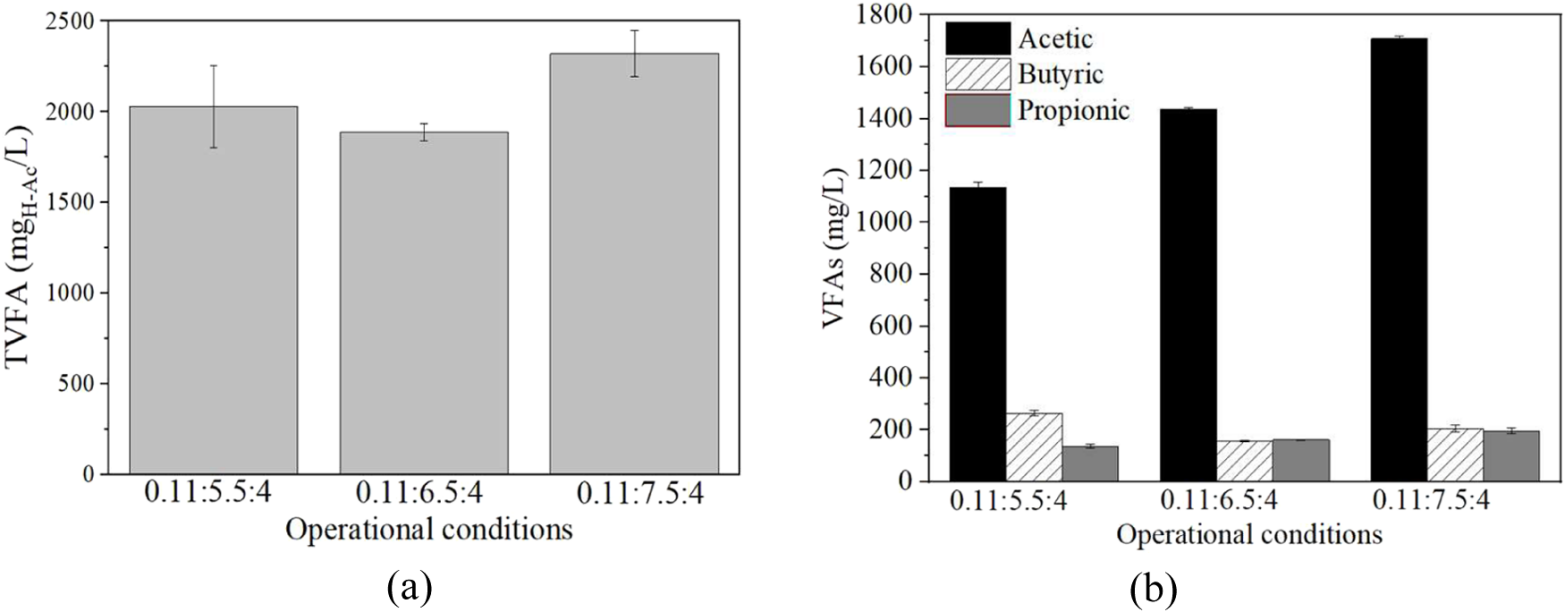
(a) Final TVFA (mg_H‑ac_/L) and (b) their profile (mg/L) for the DF of non-pretreated under different pH conditions. VFAs: acetic acid, black; butyric acid, linear pattern; propionic acid, dark gray. All of the tests were carried out at thermophilic range (*T* = 55 °C), static conditions, IS = 0.11, and SL = 4% w/v.

It has been widely reported that pH values close to 7 lead to higher dark fermentation yields as long as the reactivation of the methanogenic population is avoided.^37,38^

Accordingly, the highest H_2_ and TVFA yields were obtained at pH 7.5 (Table 2). It is in concordance with previous DF studies on other types of brown macroalgae.^39^ Consequently, IS proportion of 0.11 and a pH value of 7.5 were set for the subsequent dark fermentation trials.

#### 3.2.3 Effect of the Solids Loading (SL) in Algal Suspension

The H_2_ accumulated production was slightly increased with the increase of seaweed concentration in the substrate (Supporting Information: Figure S3). However, looking at the different seaweed concentration values presented in Table 2, it can be seen that as the alga concentration in the substrate increases, the yield progressively decreases. This reduction is more pronounced in the last two conditions studied (8% and 10% w/v), obtaining 1.5 times more in TVFA, with around 71 ± 2% of acetic acid production (Figure 4a,b), and TVFA yields of 45 ± 4.17 and 41.8 ± 1.19 mg_H–Ac_/g_biomass_, respectively (Table 2), whereas the first two conditions are quite similar in terms of TVFA yield (Table 2), reaching values of 64.5 ± 3.53 and 65.2 ± 12.3 _mgH‑Ac_/g_biomass_, respectively.

**Figure 4:**
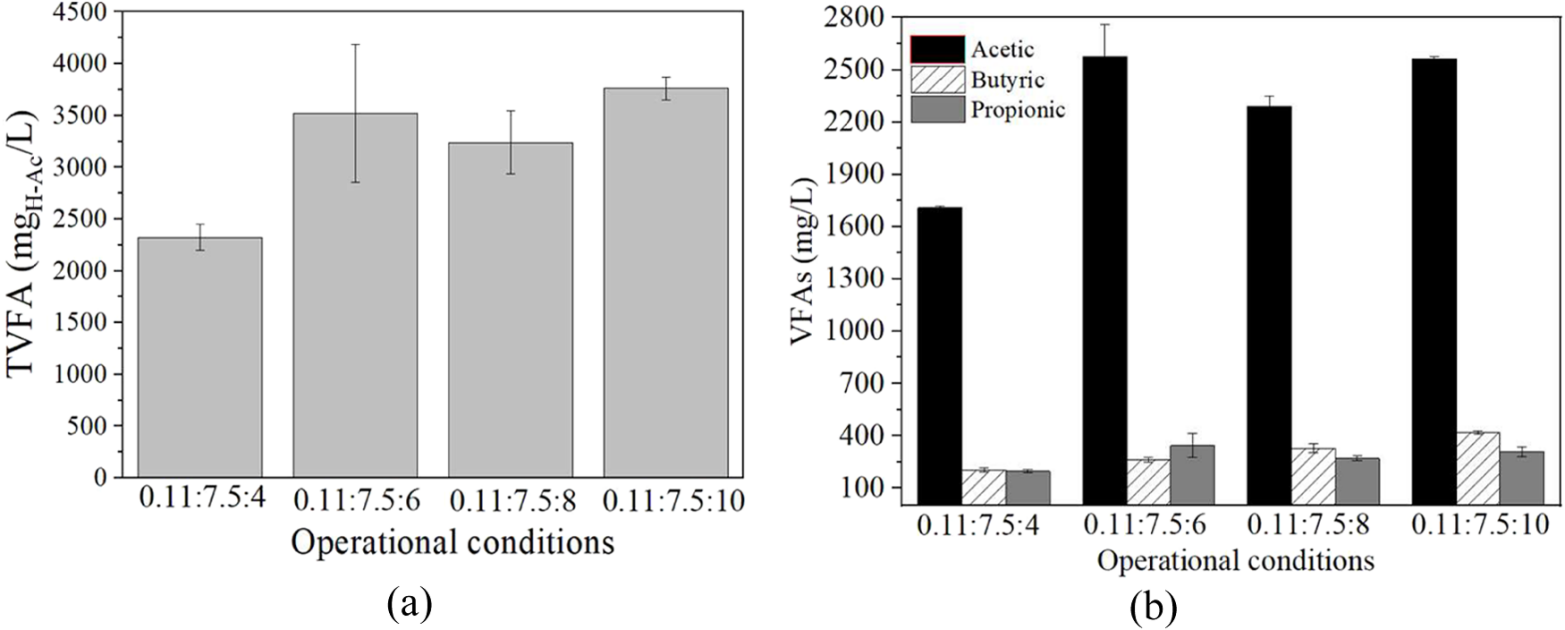
(a) Final TVFA (mg_H‑ac_/L) and (b) their profile (mg/L) for the DF of non-pretreated under different SL conditions. VFAs: acetic acid, black; butyric acid, linear pattern; propionic acid, dark gray. All of the tests were carried out at thermophilic range (*T* = 55 °C), static conditions, IS = 0.11, and pH = 7.5 w/v.

The lower yields with an increasing algal concentration may be due to an excess of available organic matter for the acidogenic microorganisms to digest. This results in a partial substrate inhibition of dark fermentation.^40,41,37^

Thus, the selection of 4 or 6% w/v seaweed concentration depends on the process to be carried out; for example, if the aim is to digest as much alga as possible, then the highest concentration may be the best option. However, if the goal is to obtain the highest amount of VFA and H_2_ from the minimum algal biomass, as in this study, 4% w/v constitutes a better option.

For this reason, in addition to the fact that H_2_ production yields are slightly higher for the 4% (w/v) condition, this concentration was established for the subsequent tests. In this sense, as the total solids content of this alga is 86.8%,^24^ the proper TS of the substrate (mg/L) would be 31.2 g_TS_/L. This result agrees with the conclusions obtained by Kim et al.,^39^ who determined an optimal range of 10–30 g_TS_/L for DF experiments.

### 3.3 Effect of Biological Pretreatment

The results achieved in the DF trials, regarding H_2_ and VFA production per gram of algal biomass, are shown in Table 3. Data are given for both biologically pretreated seaweed by and non-pretreated biomass. These results correspond exclusively to the algal biomass contribution as the contribution owed to the inoculum control has been subtracted.

**Table 3:** Results Obtained in the DF Tests Applied to Biologically Pretreated for All of the Studied Conditions over Inoculum Control, Using a Significance *p* < 0.05^a^

condition	ΔsCOD/initial vs (mg O_2_/L)/(g vs/L)	TVFA yield (mg_H–Ac_/g_biomass_)	H_2_ yield (mL_H_2__/g_biomass_)
non-pretreated	63.7 ± 8.16	65.3 ± 0.60	0.85 ± 0.42
5d 10^7^	112 ± 25.5	147 ± 3.33	3.51 ± 1.90
8d 10^7^	224 ± 25.5	133 ± 7.60	9.49 ± 2.00
5d 10^8^	128 ± 4.67	211 ± 1.25	3.49 ± 0.62
8d 10^8^	48.4 ± 17.0	82.2 ± 4.55	3.27 ± 1.25

a DF operational condition: 0.11:7.5:4.

#### 3.3.1 Organic Matter Solubilization

Algal biomass degradation is shown in Table 3 by ΔsCOD/initial vs values under each DF condition. As expected, the solubilization value increases after the DF in all of the studied conditions because of the organic matter solubilization during the degradation of algal biomass. When observing the initial sCOD values (Figure 5(a)), those corresponding to the 8-day pretreatment conditions are considerably lower than those of 5-day pretreatment. This is caused by organic matter consumption by during its growth on the macroalga biomass during the biological pretreatment. Therefore, the longer the biological pretreatment lasts, the greater the amount of algal organic matter consumed by the fungi.

**Figure 5:**
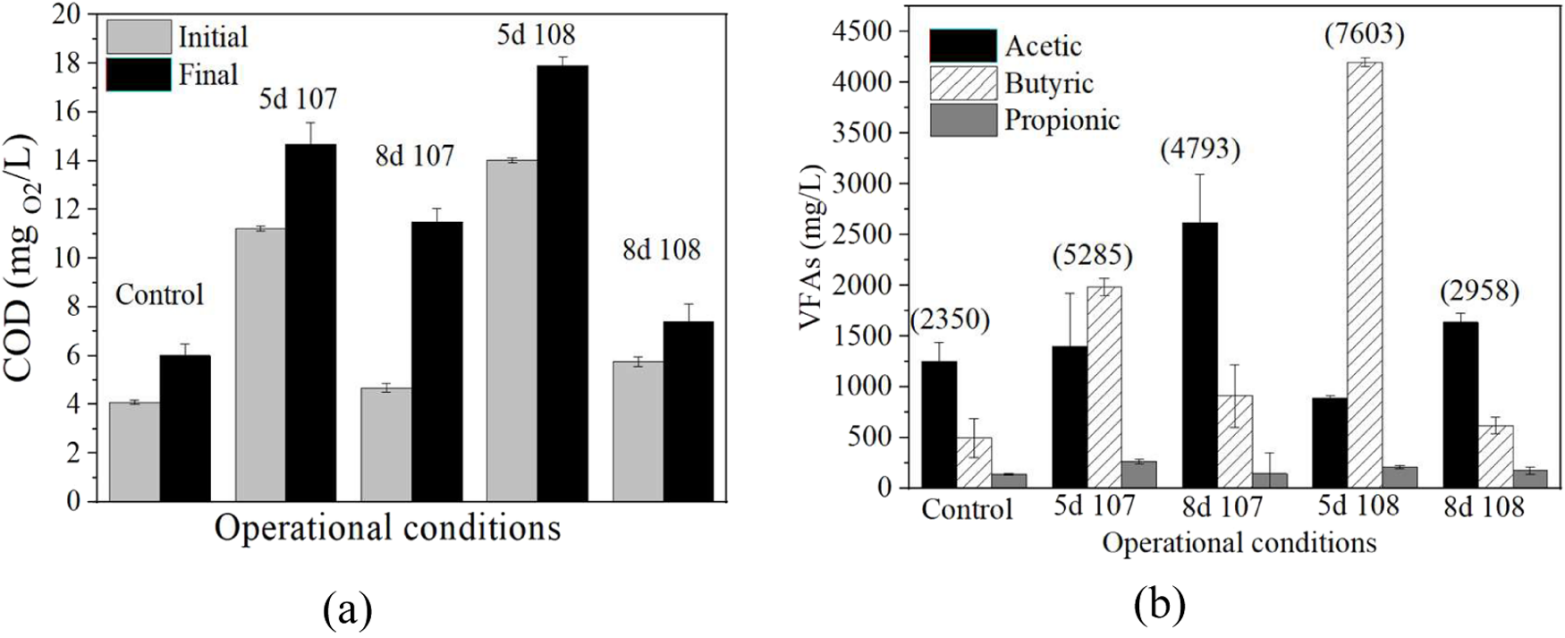
(a) Initial and final sCOD values after DF of biologically pretreated . Samples: initial values, light gray bars; final values, black bars. (b) Final TVFA (mg_H‑ac_/L) and their profile (mg/L) for the DF of pretreated at different times and in different inoculum sizes. TVFA: acetic acid, black; butyric acid, linear pattern; propionic acid, dark gray. DF operational condition: 0.11:7.5:4.

This consumption of dissolved organic matter by also explains the higher ΔsCOD values at the end of dark fermentation in the 8d 10^7^ condition pretreatment, due to higher levels of organic matter solubilization when starting from less algae organic matter (Figure 5(a)). Regarding TVFA, all of the experiments start with a similar TVFA of 1669 ± 35 mg/L (data not shown). However, the accumulation of TVFA seems to increase with the strength of the pretreatment, reaching an optimum of 7603 mg _H‑ac_/L of TVFA at 5d 10^8^ condition, >5285 mg_H‑ac_/L at 5d 10^7^, and > 4793 mg_H‑ac_/L at 8d 10^7^. This behavior was not observed for 8d 10^8^, obtaining the lowest value (2958 mg/L) after application of pretreatments. In any case, the dark fermentation of pretreated seaweed accumulates more TVFA than the control samples (2350 mg/L), obtaining higher TVFA yields (Table 3). In this sense, in this work the biological SSF was successful as a pretreatment of . Due to the release of enzymes that degrade the cell wall, polysaccharides such as cellulose, laminarin, or alginates^8^ make the soluble organic matter more available for the action of acidogenic bacteria, improving the VFA production in the DF process. So, it can be concluded that the application of the biological pretreatment with led to a significant increase in the VFA production yields.

In this sense, a short time (5 days) of pretreatment favors the VFA production, reducing the final pH until 5.4 ± 0.1. This yield was the highest in 5d 10^8^ pretreatment conditions with a final VFA concentration of 7603 mg/L, reaching a value of 211 ± 1.25 mg_H–Ac_/g_biomass_ (Table 3). On the other hand, 8d pretreatments led to lower VFA accumulation, obtaining a final pH of 6 ± 0.2. In this case, as the maximum, the final TVFA concentration of 4793 mg/L was obtained corresponding to 133 ± 7.6 mg_H–Ac_/g_biomass_ at 8d 10^7^ condition (Table 3). Likewise, Figure 5(b) shows the different fatty acid profiles present in the final samples of the DF assays. Looking at this last figure, it is quite remarkable how butyric acid was the main fatty acid produced for the samples with 5 days of pretreatment, whereas acetic acid was the main fatty acid for the samples with 8 days of pretreatment. This difference in the VFA profiles obtained for different pretreatment times with could be related to the solubilized organic matter not consumed by the fungus during the pretreatment. Therefore, a longer pretreatment time (8 days) leads to the consumption of part of the solubilized organic matter, which causes a decrease in the total VFA concentration reached. Furthermore, the results indicate that the fractions consumed by the fungus when the pretreatment time is increased should mainly give rise to butyric acid in the subsequent dark fermentation. This aspect is important when the acid distribution of the dark fermentation plays an important role in subsequent biorefinery processes. This is the case of the production of bioplastics (polyhydroxyalkanoates) or the production of medium-chain fatty acids (MCFA) through the chain elongation process.^42^

In any case, the highest VFA yield (211 mg_H–Ac_/g_biomass_) was obtained using the biologically pretreated seaweed for 5 days, inoculated with 10^8^ spores/g_biomass_. This yield represents an increase of 233.5% over non-pretreated seaweed and it is considerably higher than those obtained in other studies using more aggressive conventional methods such as microwave or microwave-acid pretreatment.^12,19^

#### 3.3.2 Bio-H_2_ Yield

In Figure 6, it can also be observed that under all of the study conditions, maximum H_2_ production is reached after the first 3 days of fermentation. Moreover, no latency times were observed in H_2_ production as its volume increases progressively from the beginning. So, it can be affirmed that in all cases, algal biomass degradation was accomplished by the acidogenic inoculum on the third day. As shown in Table 3, all of the performed pretreatments have a significant positive effect on the H_2_ production during the dark fermentation. This effect is more pronounced for the pretreatment condition of 8d 10^7^, which achieves a total H_2_ volume of approximately 50 mL. For the rest of the pretreatment conditions, the total H_2_ production does not exceed 20 mL. Comparing VFA and H_2_ production yields, it can be noticed that the highest H_2_ yield was obtained for the 8d 10^7^ condition, whereas for TVFA, it was obtained through the 5d 10^8^ pretreatment condition. This mismatch between VFA and H_2_ production yields has been reported in previous studies and is usually related to dark fermentation partial inhibitions or substrate limitation.^12^

**Figure 6:**
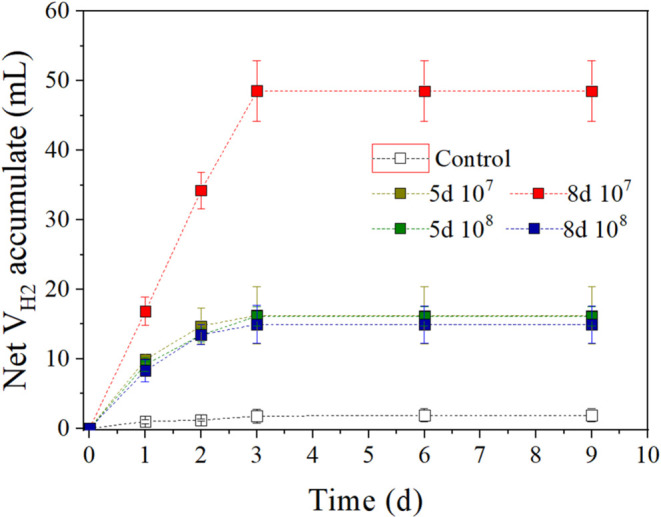
Net accumulation of H_2_ from pretreated at different times and in different inoculum concentrations.

It has been reported in the literature that the presence of polyphenols at concentrations higher than 1 g/L can cause partial inhibition of dark fermentation,^43,44^ which is not the case in this study as the maximum concentration of polyphenols measured during dark fermentation trials was approximately 160 mg/L (data not shown). Inhibition of H_2_ production has also been found to occur in the presence of excessive butyric acid.^45^ In fact, other authors have reported a large decrease in H_2_ production at pH similar to this study for butyric acid concentrations of 3 g/L.^46,47^ As can be seen in Figure 5b, butyric acid concentrations in 5d 10^8^ condition exceed 4 g/L, whereas in 8d 10^7^ condition, the final butyric acid concentrations are lower than 1 g/L. Thus, the lower H_2_ production for the 5d 10^8^ condition compared to 8d 10^7^ can be explained by a partial inhibition by the high butyric acid concentration.

This hypothesis is further supported by comparing the sCOD increases under these two conditions (Figure 5(a)). In the condition 8d 10^7^, there is a smaller increase in sCOD, despite the much higher initial and final sCOD levels. Inhibition due to higher butyric acid concentration could be responsible for this effect, despite the higher substrate bioavailability at the start of fermentation. Due to this higher bioavailability of the solubilized algal biomass at the start of the DF trials under this condition, the levels of VFA produced were also higher (133 ± 7.6 mg_H–Ac_/g_biomass_).

Thus, based on the increase in sCOD and H_2_ production yield, the 8d 10^7^ pretreatment condition results in a higher performance in terms of H_2_ production, obtaining 9.49 ± 2 mL_H2_/g_biomass_, which is increased by a factor of 9 compared to non-pretreated alga. This increase in H_2_ production is due to the high enzymatic activity generated in the biological pretreatment process, which weakens the cell-wall structures in algal biomass and then improves the dark fermentation process. Comparing this H_2_ production yield with those obtained in a previous study using microwave pretreatment at 200 °C and 20 min,^12^ an increase of 44% is observed when using biological pretreatment.

#### 3.3.3 Pretreatment Selection

Different pretreatment conditions lead to different H_2_ and VFA production yields. Hence, the selection of the most appropriate biological pretreatment condition for is intrinsically dependent on the target product to be obtained from dark fermentation. In case the aim is to obtain H_2_ from dark fermentation, the 8d 10^7^ pretreatment condition provides the highest yield. In Table 4, it can be seen that γ irradiation pretreatment of^16^ and slightly higher for showed similar H_2_ yields after heat-acid and heat-base pretreatments.^11^ The highest H_2_ yield (27.5 mL_H2_/g_biomass_) is reported for by using a combination of microwave and acid.^13^

**Table 4:** Comparison of H_2_ and VFA Productivity Results

seaweed	pretreatment condition	DF conditions	*Y*_H_2__ (mL_H2_/g_biomass_)	*Y*_VFA_ (mg_HAc_/g_biomass)_	refs
	SSF *;* 5d 10^8^	SL = 4%; *T* = 55 °C; IS = 0.11; *H* = 7.5; static	3.49	213	this work
	SSF *;* 8d 10^7^		9.49	133	
	microwave (MF 1900 MHz, 200 °C, 20 min)	SL = 4%; *T* = 55 °C; IS = 0.11; pH = 5.5 static	6.15	14.9	Fernández-Medina et al.,^12^
	microwave (MF 1900 MHz, 220 °C, 20 min)		4.18	21.5	
	microwave + acid (MF 2450 MHz, 1%H_2_SO_4_, 140 °C, 15 min)	SL = 1%; T = 36 °C; IS = 0.13; pH = 5.5 120 rpm	27.5	144	Yin and Wang^13^
	heat-base (2%, NaOH, 121 °C, 30 min)	SL = 2.4%; *T* = 36 °C; IS = 0.13; pH = 5.5 120 rpm	17.5	103	Yin and Wang^11^
	heat-acid (2% H_2_SO_4_, 121 °C, 30 min)		15.4	108	
	γ irradiation (dose 10 kGy, *T* = 25 °C)	SL = 3%; *T* = 36 °C; IS = 0.13; pH = 7 150 rpm	9.15	60	Chen et al.,^16^
	γ irradiation (dose 20 kGy, *T* = 25 °C)	SL = 3%; *T* = 36 °C; IS = 0.13; pH = 7 150 rpm	10.9	47	

Conversely, this study aims to generate VFA as biorefinery bricks for downstream processes. Bearing this objective in mind, the most favorable pretreatment condition would be the 5d 10^8^ one. The reached yield (211 ± 1.25 mg_H–Ac_/g_biomass_) is almost 1.5 times higher than that achieved with microwave-acid pretreatment (144 mg_H–Ac_/g_biomass_), which is the highest found in the literature for brown macroalgae, with the added advantage that no chemicals are used. This aspect is of great importance for the integration of the biological pretreatment in biorefineries based on VFA platforms.

## 4 Conclusions

Based on the results obtained in this study, the following conclusions can be drawn:

- The best operating conditions for the dark fermentation of the brown seaweed *Rugulopteryx okamurae* were reached at an IS ratio of 0.11, 10–90 v/v, initial pH 7.5, and solid loading in an algal suspension of 4% w/v.
- Biological pretreatment using the fungus improved the dark fermentation performance in terms of H_2_ and TVFA production.
- The pretreatment condition 8d 10^7^ achieved the highest H_2_ yield during dark fermentation (9.49 ± 2 mL_H_2__/g_biomass_), while the highest TVFA production (211 ± 1.25 mg_H–Ac_/g_biomass_) was obtained by the pretreatment condition corresponding to 5d 10^8^. This decrease in H_2_ production was due to the accumulation of butyric acid at inhibitory concentrations. This fact could be favorable for further biorefinery processes focused in TVFA production.

Taking these considerations into account, it can be concluded that biological pretreatment with the fungus improves the dark fermentation process for producing both H_2_ and TVFAs. In fact, it can achieve higher levels than some traditional, energy-intensive pretreatments such as microwave pretreatment. These results could pave the way for more sustainable alternatives in the production of biorefinery components for subsequent processes.

## Supplementary Material



## Notes

Net accumulated H_2_ generated per each round of experimental sets (PDF)

## Abbreviations

COD: chemical oxygen demand
TVFA: total volatile fatty acids
H-ac: acetic acid equivalents
