# Supplementary material for: Enhancing the Production of H2 and Volatile Fatty Acids by Fungal Pretreatment of Invasive Macroalgae () Followed by Dark Fermentation
Source: Energy Fuels. 2025 Jun 30;39(27):12962–71. doi: 10.1021/acs.energyfuels.5c01245 (PMC12258019; doi:10.1021/acs.energyfuels.5c01245)
Supplement: Supplementary file 1 [file ef5c01245_si_001.pdf]

Enhancing the production of H_2 and volatile fatty acids by fungal pretreatment of invasive macroalgae (*Rugulopteryx okamurae)* followed by dark fermentation

Pedro Fernández-Medina, Cristina Agabo, Ana Blandino, Luis I. Romero-García, Carlos J. Álvarez-Gallego.*

AUTHOR ADDRESS Department of Chemical Engineering and Food Technology, Faculty of Sciences, (Wine and Agri-Food Research Institute-IVAGRO and International Campus of Excellence-ceiA3), University of Cadiz, Republic Saharawi Avenue, P.O. Box No. 40, 11510 Puerto Real (Cádiz), Spain.

SUPPORTING INFORMATION: **Net accumulated H_2 generated per each round of experimental sets**

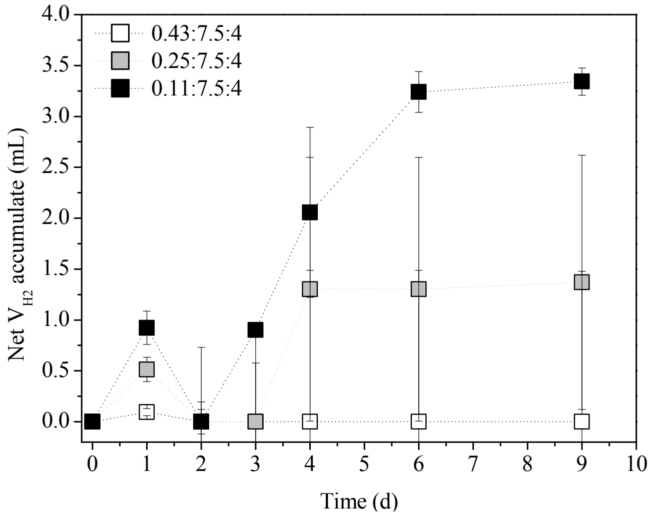

Figure S1. Accumulate H_2 produced by DF of *R. okamurae* resting the effect of the inoculum (Net V_{H2} accumulate) under different conditions of IS ratios: 0.43, white square; 0.25, light grey square; 0.11, black square.

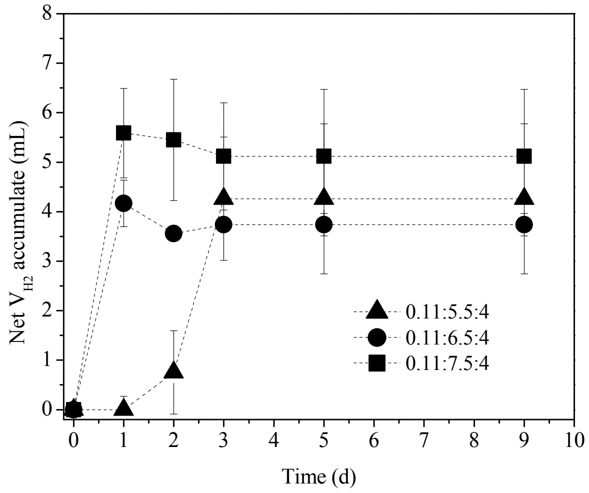

Figure S2. Accumulate H_2 produced by DF of *R. okamurae* resting the effect of the inoculum (Net V_{H2} accumulate) under different conditions of pHs: 5.5, black triangle; 6.5, black circle; 7.5, black square.

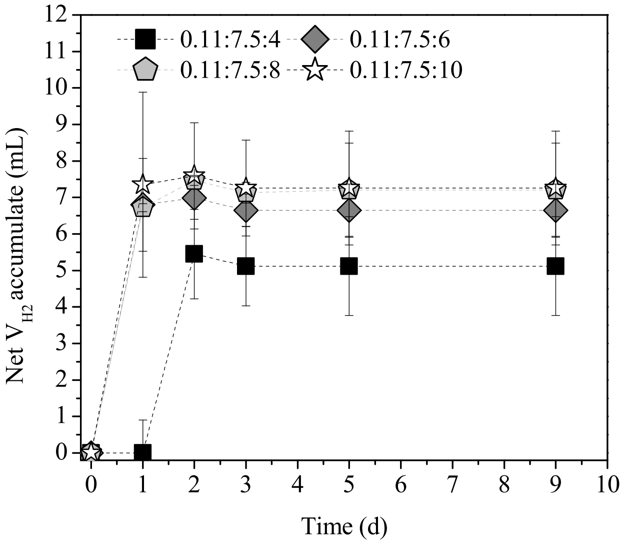

Figure S3. Accumulate H_2 produced by DF of *R. okamurae* resting the effect of the inoculum (Net V_{H2} accumulate) under different conditions of SL (% w/v) of alga in the substrate: 4%, black square; 6%, dark grey diamond; 8%, light grey pentagon; 10%, white star.
